# Synuclein Analysis in Adult *Xenopus laevis*

**DOI:** 10.3390/ijms23116058

**Published:** 2022-05-27

**Authors:** Maria Carmela Bonaccorsi di Patti, Elisa Angiulli, Arianna Casini, Rosa Vaccaro, Carla Cioni, Mattia Toni

**Affiliations:** 1 Department of Biochemical Sciences, Sapienza University, 00185 Rome, Italy; 2 Department of Biology and Biotechnology “Charles Darwin”, Sapienza University, 00161 Rome, Italy; elisa.angiulli@uniroma1.it (E.A.); carla.cioni@uniroma1.it (C.C.); 3 Department of Anatomical, Histological, Forensic Medicine and Orthopedic Sciences, Sapienza University, 00161 Rome, Italy; arianna.casini@uniroma1.it (A.C.); rosa.vaccaro@uniroma1.it (R.V.)

**Keywords:** synuclein, *Xenopus laevis*, Western blot, recombinant proteins, qRT-PCR

## Abstract

The α-, β- and γ-synucleins are small soluble proteins expressed in the nervous system of mammals and evolutionary conserved in vertebrates. After being discovered in the cartilaginous fish *Torpedo californica*, synucleins have been sequenced in all vertebrates, showing differences in the number of genes and splicing isoforms in different taxa. Although α-, β- and γ-synucleins share high homology in the N-terminal sequence, suggesting their evolution from a common ancestor, the three isoforms also differ in molecular characteristics, expression levels and tissue distribution. Moreover, their functions have yet to be fully understood. Great scientific interest on synucleins mainly derives from the involvement of α-synuclein in human neurodegenerative diseases, collectively named synucleinopathies, which involve the accumulation of amyloidogenic α-synuclein inclusions in neurons and glia cells. Studies on synucleinopathies can take advantage of the development of new vertebrate models other than mammals. Moreover, synuclein expression in non-mammalian vertebrates contribute to clarify the physiological role of these proteins in the evolutionary perspective. In this paper, gene expression levels of α-, β- and γ-synucleins have been analysed in the main organs of adult *Xenopus laevis* by qRT-PCR. Moreover, recombinant α-, β- and γ-synucleins were produced to test the specificity of commercial antibodies against α-synuclein used in Western blot and immunohistochemistry. Finally, the secondary structure of *Xenopus* synucleins was evaluated by circular dichroism analysis. Results indicate *Xenopus* as a good model for studying synucleinopathies, and provide a useful background for future studies on synuclein functions and their evolution in vertebrates.

## 1. Introduction

The synuclein (syn) family is composed of α-, β- and γ-syn, that are small soluble proteins particularly expressed in the central (α-syn and β-syn) and peripheral (γ-syn) nervous system of mammals. Syn family members were initially discovered in the cartilaginous fish *Torpedo californica* [1], and later sequenced in representative vertebrates. Comparative sequence analysis suggested these proteins were evolutionary conserved, although several differences in the number of genes encoding syn proteins have been identified in different taxa [2].

Among syn isoforms, the α-syn is especially studied as it is involved in neurodegenerative disorders (synucleinopathies) characterized by the presence of amyloidogenic α-syn inclusions that occur in neurons and glia cells [3,4,5,6,7,8]. These high social impact diseases include Parkinson’s disease (PD), dementia with Lewy bodies, PD dementia, multiple system atrophy, and several less well-characterized neuroaxonal dystrophies [1,9,10,11,12,13,14]. The direct involvement of α-syn in PD was demonstrated by the identification of point mutations in the human α-syn gene (*snca*) that results in autosomal-dominant PD [15,16,17,18,19,20,21,22,23,24,25].

Experimental evidence collected in the last 15 years support the existence of a “prion-like” mechanism in synucleinopathies, and Prusiner and co-workers defined α-syn as a new human prion [26]. Human α-syn is a natively unfolded protein of 140 aa (about 14 kDa) that can adopt different secondary structure shifting from a random conformation to α-helix or to anti-parallel β-sheet conformation when bound to phospholipid vesicles [27] or aggregated into fibrils [27,28,29,30], respectively. Furthermore, experimental evidence suggests a prion-like neuron-to-neuron spread of α-syn [31,32].

The exact physiological functions of α-syn have not yet been fully clarified, but experimental evidence demonstrate its involvement in vesicular trafficking and neurotransmitter release for its ability to bind to phospholipid vesicles assuming an α-helix conformation and to interact with the SNARE complex [33,34]. The β- and γ-syn isoforms are expressed also in peripheral tissues and the understanding of their physiological function is still quite limited.

The study of syns in non-mammalian vertebrates would help in understanding the evolution and physiological role of these proteins and in developing animal models for the study of synucleinopathies. However, information on syns of fish [2,35,36], amphibians [37,38,39,40], reptiles [41] and birds [42,43,44] is limited to a few studies.

The study of syns could take advantage of model species used in scientific research which can be easily obtained from animal facilities and whose genome has been sequenced such as zebrafish *Danio rerio* for teleosts, African clawed frog *Xenopus laevis* for amphibians or lizard *Anolis carolinensis* for reptiles. However, not all organisms are suitable for the study of the physiological expression of α-syn, such as zebrafish, which lacks the *snca* gene [45,46,47].

*Xenopus laevis* (hereafter in this article referred as “*Xenopus*”) has been used as a model organism in various scientific research fields including neuroscience [48,49,50,51,52], and it could be a good model for the study of synucleinopathies. The knowledge of syns in *Xenopus* is limited to the analysis of the gene expression of the three isoforms during embryogenesis [39], and to the attempt to analyse β-syn protein expression [37]. Since synucleinopathies occur in adulthood in humans and their onset is related to the accumulation and spread of misfolded α-syn proteins, it is important to study syn proteins in the adult stage. For these reasons, in the present work, α-, β- and γ-syn gene expression was analysed in the central nervous system (CNS) and in the main organs of adult *Xenopus* by qRT-PCR. Furthermore, recombinant α-, β- and γ-syns were produced to investigate the secondary structure by circular dichroism and to test the ability of a commercial antibody against human α-syn to recognize and discriminate *Xenopus* α-syn from β- and γ-syn by Western blot analysis. Finally, the same antibody was used in Western blot and immunohistochemical experiments to analyse α-syn expression and distribution in *Xenopus* samples.

## 2. Results

The information on the nucleotide and amino acid sequences of *Xenopus* syns currently present in the NCBI database consisted of V10.1 Primary assembly. Unlike mammals in which three syn genes (*snca*, *sncb* and *sncg*) are present, six genes (two for each isoform) were identified in the *Xenopus* genome. This depends on the tetraploid condition of this species, which is characterized by L and S homologous chromosomes [53]. The genes coding for α-syn are located on chromosomes 1L (*snca L*, gene ID: 380315) and 1S (*snca S*, gene ID: 100037108), for β-syn on chromosomes 3L (*sncb L*; gene ID: 495448) and 3S (*sncb S*; gene ID: 443875) and for γ-syn on chromosomes 7L (*sncg L*; gene ID: 432294) and 7S (*sncg S*; gene ID: 380522). All the mRNA sequences of *Xenopus* syns available in NCBI were complete, except for S α-syn, which is missing the initial part of the coding sequence (Figures S1–S3). The comparative analysis showed a good degree of homology between the L and S mRNA coding sequence of the same isoform, and consequently of their amino acid sequence (Figures S1–S3 and Table 1).

**Figure 1:**
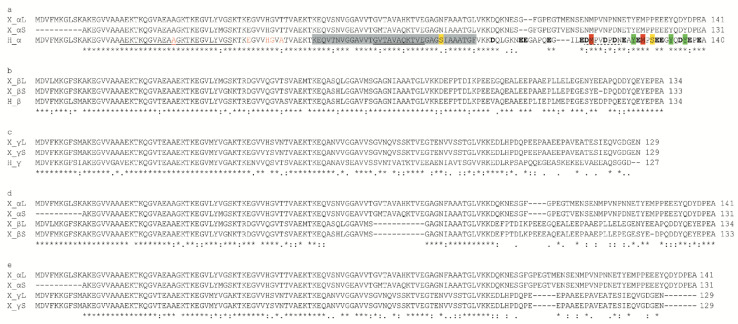
Alignment of syn amino acid sequences. Comparisons among human and *Xenopus* α- (**a**), β- (**b**) and γ- (**c**) syns, among *Xenopus* α- and β-syns (**d**), and among α- and γ-syns (**e**). (**a**) Conserved repeats of the apolipoprotein lipid-binding motif [EGS]-KT-K-[EQ]-[GQ]-V-XXXX are underlined. The non-amyloid-component (NAC) region of α-syn, the phosphorylatabletyrosines and serines are highlighted in grey, green and yellow, respectively. The methionines representing binding sites for Mn(II) and other metals are highlighted in red. Negative amino acids in the CT region are indicated in bold. The amino acids that in humans are involved in the pathological mutations linked to Parkinson’s disease are shown in red. The aa stretch GVTAVAQKTVE that is directly involved in the formation of human amyloid fibrils is double underlined. The sequences were aligned with Clustal Omega. Asterisks indicate identity of amino acids; double dots indicate amino acids with the same polarity or size; dots indicate semiconserved substitutions. The epitope recognized by the ab27766 antibody is dotted underlined.

The α-, β- and γ-syns showed a high homology with their respective human isoforms (Figure 1a–c), as expected from previous studies [39]. Key amino acids, such as the apolipoprotein lipid-binding motif ([EGS]-KTK-[EQ]-[GQ]-V-XXXX), most of the phosphorylatable tyrosines and serines, and two methionines representing binding sites for Mn and other metals, are conserved in the *Xenopus* and human α-syn (Figure 1a). Furthermore, the C-terminal region of *Xenopus* α-syn contains most of the negative amino acids present in human α-syn. Interestingly, both L and S α-syn contain a threonine instead of alanine in position 53 of the human protein (Figure 1a), and this substitution may be biologically relevant since A53T mutation in humans is linked to PD [54].

As in human syns, a high homology of the N-terminal region was observed among *Xenopus* α-, β- and γ-syns, while a lower degree of homology was observed in the C-terminal region (Figure 1d,e).

### 2.1. Synuclein Gene Expression

**Figure 2:**
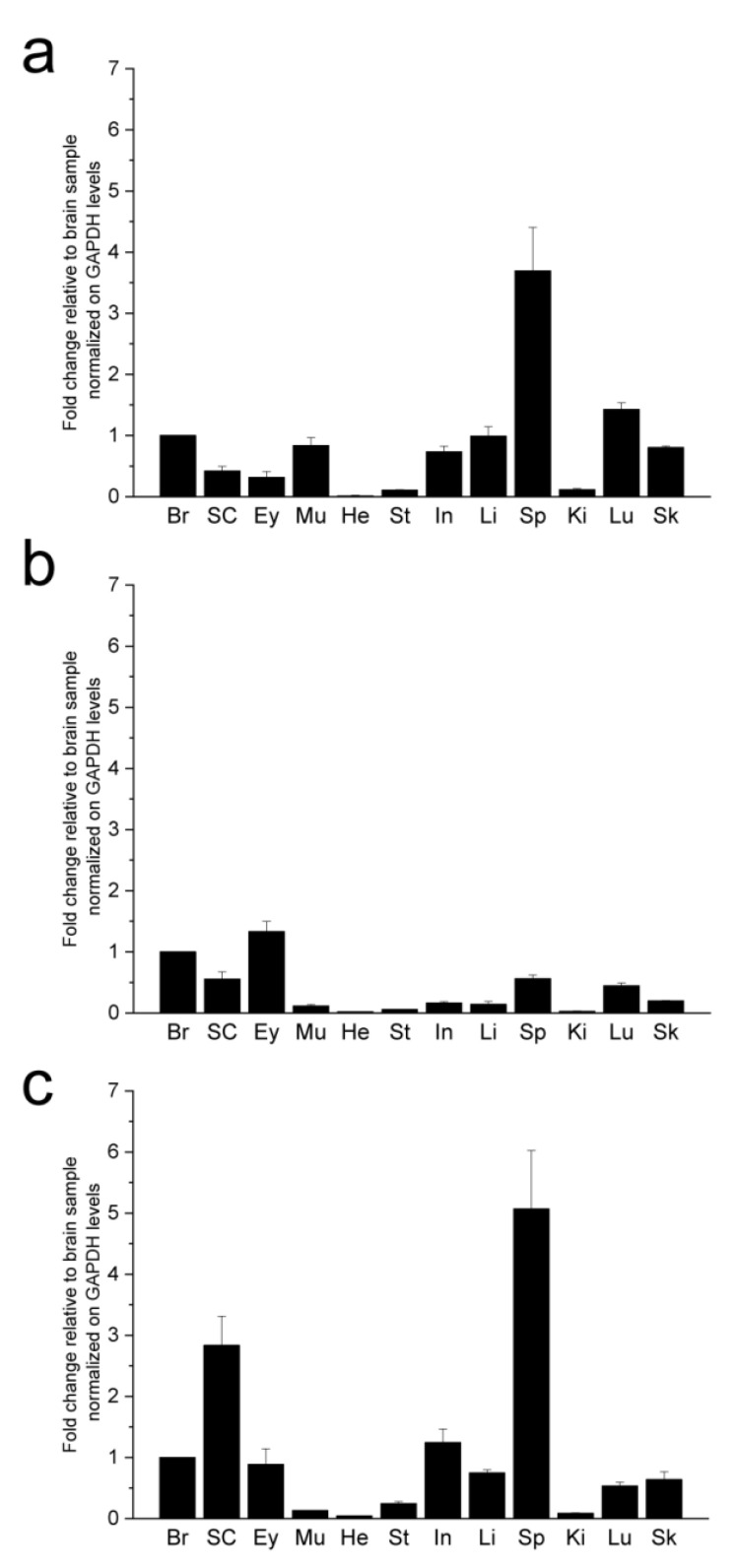
Syn gene expression in the major organs of adult *Xenopus*. qRT-PCR analysis of α- (**a**), β- (**b**) and γ- (**c**) syn gene expression in the main organs of adult *Xenopus*. Expression levels were normalized against GAPDH and expressed as fold change relative to brain sample. Br: brain, SC: spinal cord, E: eye, Mu: muscle, He: heart, St: stomach, In: intestine, Li: liver, Sp: spleen Ki: kidney, Lu: lung, Sk: skin.

Syn gene expression was evaluated in the major organs of the *Xenopus* by qRT-PCR (Figure 2). The availability of the mRNA sequences coding for syn isoforms allowed us to design specific primers able to discriminate α-, β- and γ-syn expression (Table 2). The three isoforms were expressed both in nervous (brain, spinal cord and eye) and non-nervous (intestine, kidney, liver, lung, muscle, skin, stomach, heart and spleen) organs (Figure 2). In the nervous system, syns showed differences in the relative expression pattern as α-syn was more expressed in the brain, β-syn in the eye and γ-syn in the spinal cord. Among non-nervous tissues, high levels of α-syn mRNA were detected in the spleen and lung, discrete levels in the muscle, intestine, liver, and skin, whereas low or undetectable levels were detected in the kidney, stomach and heart. Discrete expression of β-syn mRNA was detected in the spleen and lung, and γ-syn expression was particularly high in the spleen and discrete in intestine, liver, lung and skin (Figure 2).

### 2.2. Synuclein Recombinant Protein Production

**Figure 3:**
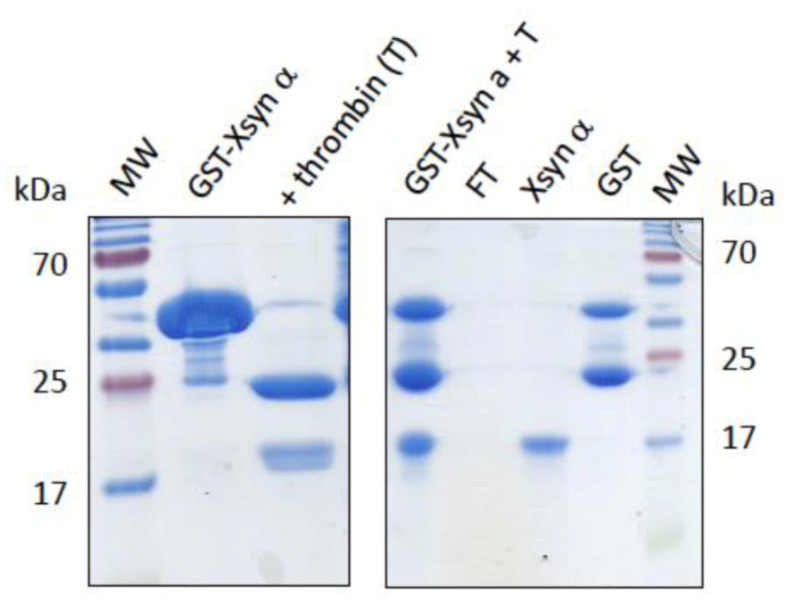
SDS-PAGE analysis of purified recombinant *Xenopus* α-syn (Xsynα). (**Left panel**), purified GST-Xsynα before and after treatment with thrombin. (**Right panel**), GSH-Sepharose chromatography fractions: GST-Xsynα treated with thrombin, flow-through (FT), Xsynα and GST recovered in the wash and GSH-eluted fractions, respectively; MW: molecular weight markers.

The coding sequences of the three *Xenopus* syn isoforms were cloned in pGEX-2T and the recombinant GST-syn fusion proteins were expressed in *E. coli* BL21 (DE3) and purified by affinity chromatography on GSH-Sepharose. The GST-syn fusion protein was recovered with high yield and high purity (Figure 3, left panel). The GST tag was removed by treatment with thrombin followed by separation on GSH-Sepharose and recovery of pure syn in the wash fraction. Figure 3 shows representative results obtained for α-syn; all syn isoforms were obtained and purified in the same conditions with similar yield and purity.

### 2.3. Alpha Synuclein Antibody Validation

Antibodies able to recognize and discriminate the different syn isoforms are needed to study their expression and distribution. Given the high level of homology between human and *Xenopus* α-syn (Figure 1a–c), commercial antibodies against human α-syn might be successfully used also in the *Xenopus*. However, the selection of suitable commercial antibodies specific for α-syn is not easy due to the high homology of the first 100 amino acids of *Xenopus* syns (Figure 1d,e) and to the low homology between the C-terminus of human and *Xenopus* α-syn (Figure 1a).

**Figure 4:**
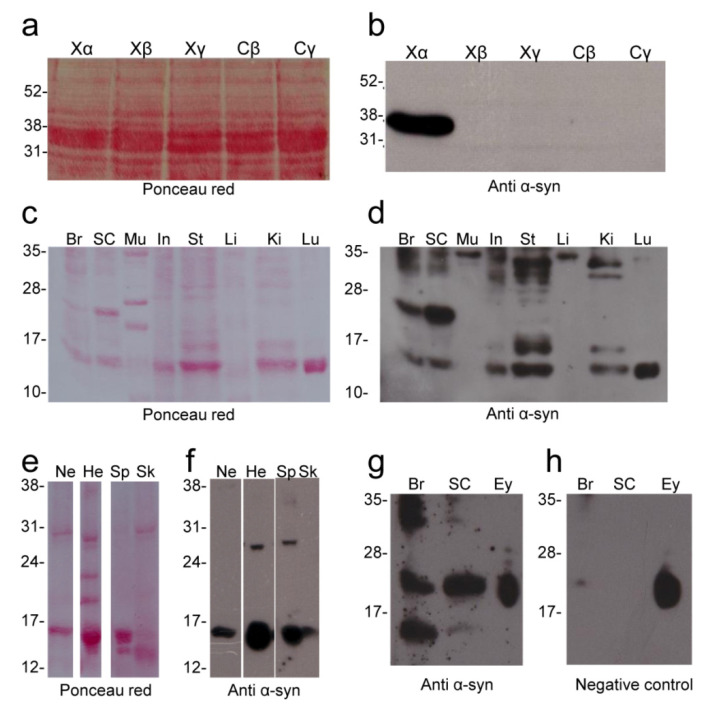
Western blot analysis of α-syn expression by ab27766 antibody. Validation of the antibody on *Xenopus* (Xα, Xβ, Xγ) and *Cyprinus carpio* (Cβ and Cγ) recombinant syns (**a**,**b**). α-syn expression in the main *Xenopus* organs (**c**,**h**). Red ponceau staining is shown in (**a**,**c**,**e**). α-syn immunolabelling in (**b**,**d**,**f**,**g**). Negative control (primary antibody omitted) in (**h**). Br: brain; Ey: eye; He: heart; In: intestine; Ki: kidney; Li: liver; Lu: lung; Mu: skeletal muscle; Ne: nerve; SC: spinal cord; Sk: skin; Sp: spleen; St: stomach; Xα, Xβ and Xγ: *Xenopus* recombinant α-, β- and γ-syn, respectively; Cβ and Cγ: carp recombinant β- and γ-syn, respectively.

In this work, *Xenopus* recombinant α-, β- and γ-syns were used to test, by Western blot, the ab27766 (abcam, UK) antibody directed against the 115–122 amino acid region of human α-syn (Figure 1a,b). The ab27766 antibody intensely labelled a band corresponding to the recombinant α-syn fused to glutathione S-transferase (GST, GST-tagged α-syn), while it does not recognize *Xenopus* and carp β- and γ-syn (Figure 4b). The results identify this antibody as suitable for the recognition and discrimination of α-syn in *Xenopus*.

### 2.4. Alpha Synuclein Protein Expression

The same antibody (ab27766) was used to verify the α-syn protein expression in main *Xenopus* organs by Western blot (Figure 4b–h). The main purpose of these analyses was to confirm and corroborate the data obtained from qRT-PCR experiments demonstrating the effective expression of α-syn protein in the brain and other organs analysed.

An immunolabelled band at 14–15 kDa (corresponding to the α-syn predicted MW) was detected in most of the organs examined (brain, spinal cord, nerve, intestine, stomach, kidney, lung, heart, spleen and skin) (Figure 4d,f,g). Moreover, immunolabelled bands at higher molecular weight that could correspond to oligomers were detected. An intense immunolabelled band was also detected at 26–27 kDa in the CNS (brain and spinal cord) and in the heart and spleen. Bands at higher molecular weight (range 31–35 kDa) were also detected in most organs. Interestingly, the skeletal muscle and liver showed only an immunolabelled band at 35 kDa. In the eye, no immunolabelled bands were observed at 14–15 kDa. It was not possible to establish the presence of any bands at 26–27 kDa in the eye due to the presence of intense non-specific immunolabelling in the range 20–27 kDa, as shown by negative control in which the primary antibody was omitted (Figure 4h).

### 2.5. Immunohistochemical Analysis

**Figure 5:**
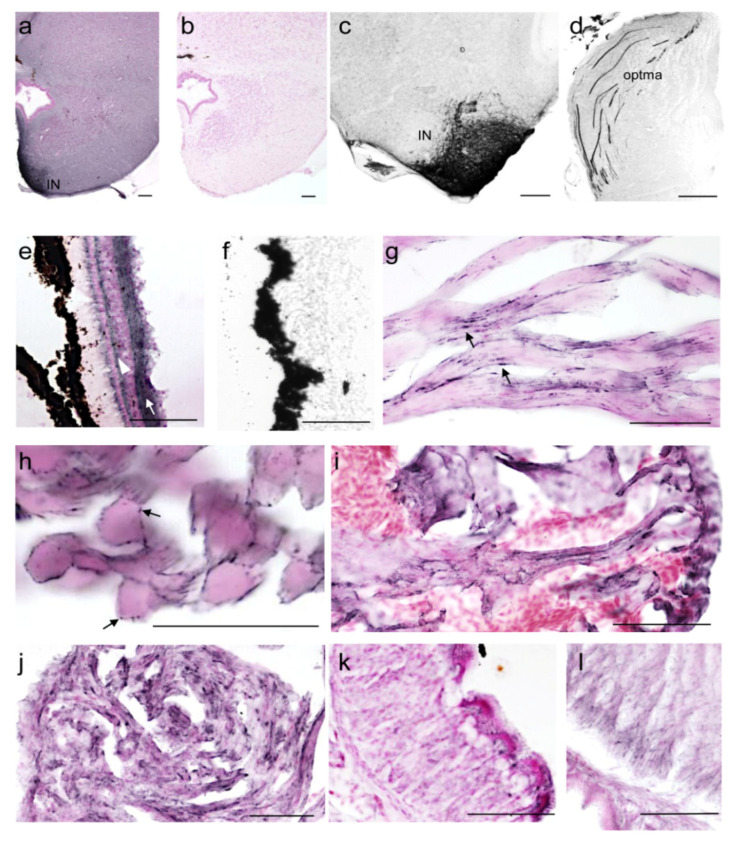
Immunohistochemical analysis of the α-syn distribution. *Xenopus* brain coronal sections (**a**–**d**). Strong α-syn immunoreactivity was found in the *interpeduncular nucleus* (**a**,**c**) and in the visual projections, *tractus opticus marginalis* (**d**). Retina (**e**,**f**). The strongest α-syn immunoreactivity was found in the thick inner plexiform layer (white arrow) and in the outer plexiform layer (white arrowhead) (**e**). No immunoreactivity was found in control sections (**b**,**f**). The α-syn immunoreactivity was found in motor nerve endings within skeletal muscle (longitudinal (**g**), and transverse section (**h**), arrows) and heart muscle (**i**,**j**). α-syn immunolabelled nerve fibres were found also within all layers of the stomach wall (**k**,**l**). Some sections have been counterstained with Nuclear Fast Red Solution. IN: *interpeduncular nucleus*; optma: *tractus opticus marginalis*. Bar = 100 µm.

The ab27766 antibody was also tested in a preliminary immunohistochemical experiment (Figure 5). The results showed α-syn positive soma and nerve fibres in the brain (Figure 5a–d). By way of example, positive neurons were detected in the *interpeduncular nucleus* (Figure 5c) and immunolabelled fibres in the *tractus opticus marginalis* (Figure 5d). Moreover, α-syn immunostaining was detected in the inner and outer plexiform layer of the retina (Figure 5e). The immunohistochemical analysis of skeletal muscle (Figure 5g,h), heart (Figure 5i,j) and stomach (Figure 5k,l) samples revealed α-syn immunolabelling mainly limited to the nerve fibres and neuromuscular junctions. Based on these results, it will be possible in future studies to carry out an in-depth study of α-syn distribution in the CNS and organs of *Xenopus*.

### 2.6. Structural Characteristics of Xenopus Synucleins

**Figure 6:**
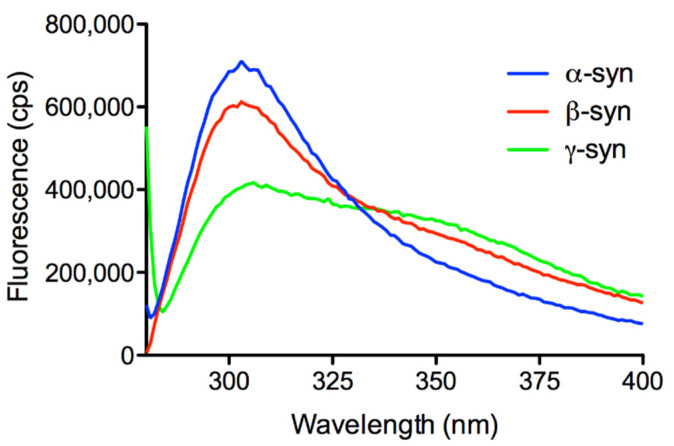
Fluorescence spectra of purified recombinant *Xenopus* syns. Protein concentration was 0.11 mg/mL for α- and β-syn, and 0.24 mg/mL for γ-syn.

Spectroscopic analyses of *Xenopus* recombinant syns were performed to evaluate the properties of the purified proteins. The fluorescence spectra presented in Figure 6 were characterized by tyrosine emission due to the lack of tryptophan residues in the amino acid sequence of all isoforms of the protein. Four tyrosines are present in α- and β-syn sequences and one in γ-syn, all of which are conserved in the corresponding human isoforms. The α- and β-syn spectra were very similar and presented an intense peak at about 302 nm, typical of tyrosine. The γ-syn spectrum displayed a broader peak with a shoulder between 340–360 nm, which was weakly visible also in the β-syn spectrum. This feature has been suggested to depend on the tyrosinate form of this residue due to the possible proton transfer to nearby acidic residues [55].

**Figure 7:**
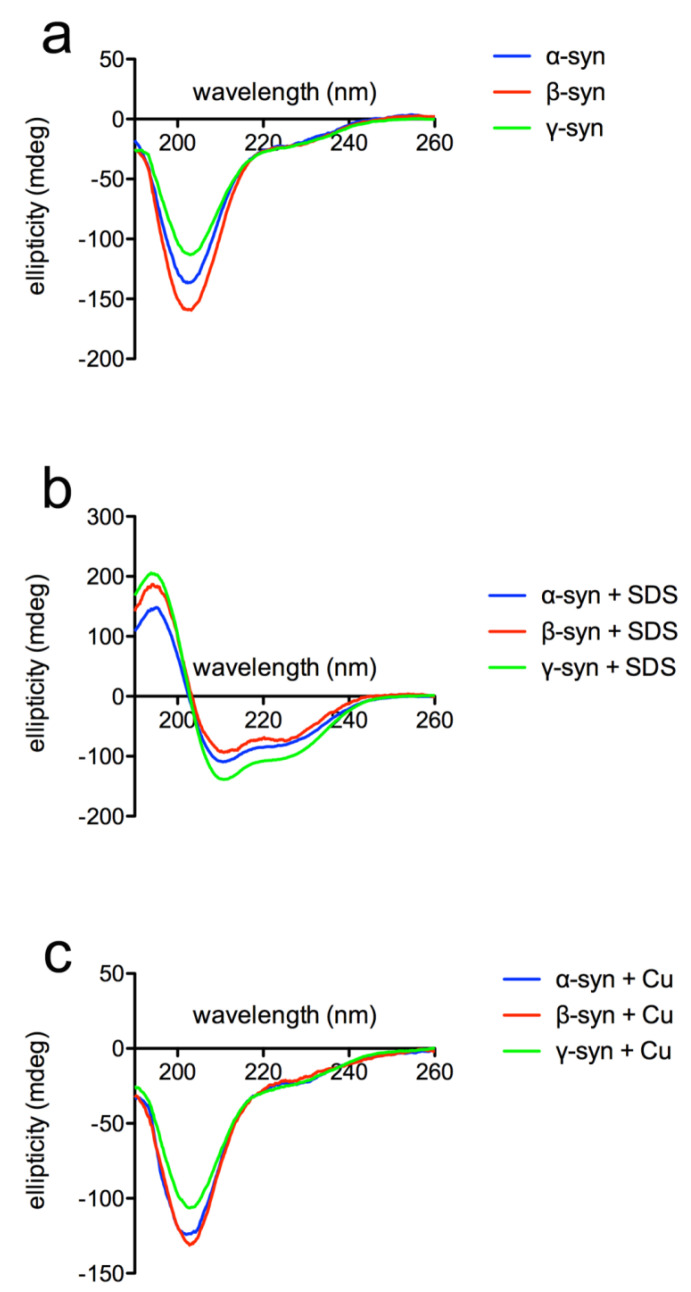
CD spectra of purified recombinant *Xenopus* syns. The proteins were diluted in 10 mM potassium phosphate buffer pH 7, containing 50 mM Na_2_SO_4_ (**a**); SDS was added at 10 mM (**b**), while CuSO_4_ was added at 100 µM final concentration (**c**). The spectra are normalized for protein concentration.

Far-UV CD spectra of the proteins confirmed the predominantly unfolded state of all the three isoforms (Figure 7a) with a typical intense negative peak at about 200 nm. The anionic detergent SDS has been used to test the interaction of syns with micelles/membranes and to evaluate the conformational transition from unfolded to α-helical secondary structure [56]. After the addition of SDS, the CD spectra clearly showed a shift towards an α-helical structure (Figure 7b), in line with previous reports on mammalian syn isoforms [57,58].

Syns have the ability to bind metals such as copper, which may be physiologically relevant [59,60,61,62]. The addition of copper did not change the CD features of all three *Xenopus* syn isoforms (Figure 7c). These results suggest that there are no gross differences among syn isoforms, at least as far as secondary structure and response to membranes and copper are concerned. The presence of the PD-linked variant A53T in *Xenopus* α-syn does not appear to impact the secondary structure of the protein. It should be reminded that threonine in position 53 occurs naturally in most mammalian species [63] and its role in toxicity can be significantly altered/decreased by combination with other substitutions. The results of the CD analyses indicate that *Xenopus* syns appear to be similar to mammalian syns as far as their behaviour in the presence of SDS or copper is concerned.

**Figure 8:**
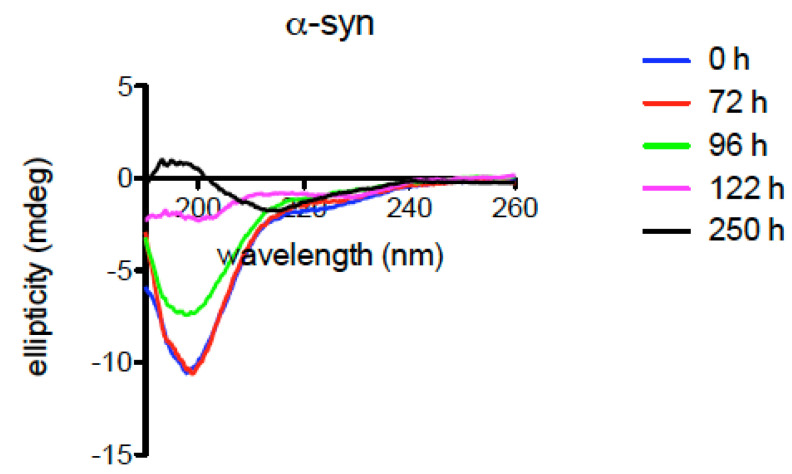
CD spectra of *Xenopus* α-syn. The protein (10 μM) in 10 mM potassium phosphate buffer pH 7, containing 50 mM Na_2_SO_4_, was incubated at 37 °C for the specified times.

To further investigate the properties of *Xenopus* α-syn, the protein was incubated at 37 °C for 250 h, and CD spectra were recorded at the indicated times (Figure 8). The results demonstrate progressive conversion from the unfolded state to a β-sheet secondary structure, typical of aggregated syn (Figure 8). Further experiments are needed to fully evaluate the propensity of *Xenopus* syn isoforms to aggregate and form fibrils.

## 3. Discussion

Syn proteins are expressed in representatives of all vertebrates, although differences in the number of coding genes and isoforms can be identified, especially in teleosts [2], in which a major variability in the number of syn isoforms has been observed. This has been attributed to the whole genome duplication that occurred in the ray-finned fishes (Osteichthyes, Actinopterygii) about 230 million years ago, which was followed by the subsequent loss of some duplicated genes, depending on the species [2]. For example, the zebrafish *Danio rerio*, a teleost model organism in neuroscience, possesses a gene for β-syn, two genes for γ-syn (γ1 and γ2), but no gene for α-syn [2].

Among amphibians, *Xenopus* is a model organism widely used in scientific research. It is an allotetraploid organism with 36 chromosomes (2n = 36), which is believed to derive from the interspecific hybridization of diploid progenitors with 2n = 18, which occurred 17–18 million years ago [53]. The chromosomes deriving from each diploid progenitor are referred to as L (longer) and S (shorter) due to their different lengths. Due to the tetraploid condition, most *Xenopus* genes have two copies, defined as L and S. Consequently, six genes coding for syns (two genes for each isoform) have been sequenced in the *Xenopus* genome. The amino acid sequences of the two proteins encoded by each pair of L and S genes showed a high homology degree, suggesting that both homologous isoforms may have similar physiological functions, although specific studies must be carried out to verify whether L and S isoforms can be involved in different physiological processes.

*Xenopus* and human syns show a good degree of homology, suggesting that the conservation of the amino acid sequences may correspond to conserved cellular and physiological roles. In particular, the conservation of the apolipoprotein lipid-binding motif, putative sites of phosphorylation or interaction with metals, suggests that α-syn can have similar biological functions in *Xenopus* and humans. However, some differences that could impact the properties of the protein were observed. In fact, the comparison between human and *Xenopus* α-syn showed the A53T substitution in both *Xenopus* L and S proteins. This is not surprising since a threonine at position 53 occurs naturally in α-syn of most animals [54,63]. However, in humans and other mammals, the physiological α-syn isoform has an alanine at position 53, and the A53T substitution is associated with familial forms of PD. Moreover, the A53T α-syn has been shown to exhibit a greater tendency to aggregate than the physiological isoform [64,65]. Since synucleinopathies are related to α-syn aggregation and accumulation in the CNS, it is believed that the presence of the A53T substitution may facilitate these processes and PD onset. In this respect, *Xenopus* can be a potential model of synucleinopathy since it physiologically expresses α-syn with the A53T substitution, and the tetraploid condition could increase the intracellular amount of α-syn, both conditions potentially favouring protein aggregation. CD analyses confirm that *Xenopus* α-syn converts to a β-sheet secondary structure, indicative of aggregation. Further studies are needed to investigate in detail the tendency of *Xenopus* α-syn to aggregate in vitro and in vivo.

Studies on syn gene expression during *Xenopus* development have reported that α-syn gene (*snca*) is expressed since the gastrula stage and it is localized in the olfactory placode, brain, otic vesicle, branchial arches, and somites in the bud stage [39], thus suggesting a wide distribution in the embryos. These results have been confirmed in adult *Xenopus* by present qRT-PCR results, revealing α-syn gene expression in most of the organs analysed. High levels were detected in the CNS (brain and spinal cord), eye and spleen, consistent with results obtained in mice [66]. Discrete levels were also observed in the lung, skin, liver, skeletal muscle and intestines. The α-syn expression in skeletal muscle and lung was consistent with results obtained in humans [67], whereas α-syn expression in *Xenopus* heart appeared lower compared to lizards [41] and humans [67].

The β-syn expression in humans has a more restricted distribution compared to α-syn [67,68,69], and this was also evident in *Xenopus* embryos where β-syn gene expression was limited mainly to the brain and spinal cord at the tail bud and tadpole stages [39]. Present results demonstrate that β-syn expression remains limited to the brain, spinal cord, eyes, spleen, lung and skin, even in the adult frog. In contrast, moderate β-syn levels were observed in a higher number of organs in the lizard *Anolis carolinensis* [41].

The γ-syn gene expression in *Xenopus* embryos became detectable at the gastrula stage and was mainly localized in cranial nerves, cranial and dorsal root ganglia and pineal gland in the tail bud stage [39]. In adult *Xenopus*, high levels of γ-syn expression are detected in the CNS (brain and spinal cord), eye, spleen, liver, intestine, lung and skin by qRT-PCR analysis, whereas low levels are detected in muscle and the heart. The reduced syn expression in the heart is consistent with results obtained in the lizard [41].

As is well known, the analysis of syn protein expression requires specific antibodies capable of discriminating one specific isoform from the others. For this purpose, in the present work we have produced *Xenopus* α-, β- and γ-syn recombinant proteins that were used in Western blot experiments to test the ability of the ab27766 monoclonal antibody against mammalian α-syn, to recognize *Xenopus* α-syn and not β- and γ-syns. The same antibody was then used to verify α-syn expression in the main organs of adult *Xenopus*. The ab27766 antibody (abcam, UK) tested by immunohistochemistry on nervous and non-nervous tissues has proved to be a suitable tool to localize *Xenopus* α-syn. Western blotting showed the presence of a band at about 15 kDa (consistent with α-syn predicted molecular weight, MW) in most of the organs examined, confirming the wide distribution of α-syn, consistent with results in humans and monkey, showing that α-syn is highly enriched in the brain, but also widely distributed in other tissues, especially at fetal stages [70,71]. Immunolabelled bands were also detected at higher molecular weight that could represent α-syn dimers or oligomers. In interpreting Western blot results, it must be considered that two different genes, encoding L- and S-α-syn, respectively, are present in the *Xenopus* genome. The complete L-α-syn sequence encoding a protein with a predicted MW of 14.7 kDa is available in the current genome assembly, while the S-α-syn sequence is incomplete, and consequently its MW cannot be accurately estimated. Furthermore, both L- and S-α-syn are characterized by the A53T substitution, which could make the proteins more prone to aggregate into oligomers. These features could explain the presence of immunolabelled bands with MW higher than 17 kDa. Interestingly, an intense immunolabelled band at 26–27 kDa was observed mainly in the CNS (brain and spinal cord), that could result from the high expression of specific α-syn isoforms in these tissues. A precise analysis of α-syn expression in the eye was not possible by Western blot due to the presence of a non-specific labelling in the 20–27 kDa range. Surprisingly, the non-specific labelling was not observed in the immunohistochemical analysis of the eye. The α-syn immunolabelling was observed both in neuronal soma and nerve fibres in the CNS. In the eye, immunopositivity was observed in the inner and outer plexiform layer confirming previous studies [72], and suggesting an evolutionarily conserved role of α-syn in this organ. Immunopositive labelling for α-syn was also detected in muscle tissues. In the cross section of skeletal muscle fibres, immunolabelled spots were observed at the sarcolemmal level, suggesting α-syn localization in neuromuscular junctions. This is consistent with results demonstrating α-syn presence, especially in the postsynaptic domain of neuromuscular junctions in humans [73]. However, TEM observations are necessary to detail α-syn distribution at this level. As observed in humans [74], α-syn positive fibres were also observed in *Xenopus* stomach.

Similar to present data, previous experiments by Yuan and collaborators [37] used a *Xenopus* β-syn recombinant protein to produce a specific antibody against β-syn, through which they revealed β-syn expression in the brain but not in the heart and liver. Interestingly, these results are consistent with the scarce or absent β-syn gene expression detected in the same organs by qRT-PCR.

Human syns are natively unfolded proteins [75,76] which can undergo conformational changes following interaction with membranes [76,77], or alteration of physicochemical parameters such as temperature and pH [55]. The protein conformation strongly influences syn tendency to aggregate [55,78] and, especially for α-syn, to contribute to the onset of synucleinopathies. The production of purified recombinant proteins in *Xenopus* allowed us to begin the characterization of the structural features of amphibian syns.

In line with the high sequence homology with human syns, the fluorescence and CD data indicate general conservation of the secondary structure and response to detergents and copper. Moreover, like human α-syn, the *Xenopus* α isoform changes its conformation over time in experiments of in vitro incubation at 37 °C, converting to a β-sheet secondary structure. Overall, these results reinforce the proposition that *Xenopus* may be a good model for the study of synucleinopathies.

The availability of recombinant syns will make it possible to carry out further in-depth analyses to highlight possible subtle differences between amphibian and human syn that could corroborate the goodness of *Xenopus* as a model for the study of synucleinopathies, and provide new insights into the role and evolution of vertebrate syn proteins.

## 4. Materials and Methods

### 4.1. Animals and Sampling

Nervous (brain, eye, spinal cord and nerve) and non-nervous organs (intestine, kidney, liver, lung, muscle, skin, stomach, heart, spleen) from 14 adult individuals of *Xenopus* were used. Tissues from 12 animals were stored in RNA Later (Ambion, Austin, TX, USA) at −70 °C until they were processed for RNA or protein extraction, and those from 2 animals were fixed in PFA fixative (4% paraformaldehyde in 0.1 M phosphate buffer) and then stored at 4 °C in 0.01 M phosphate buffer (PB) containing 15% of sucrose until they were processed for immunohistochemistry.

### 4.2. qRT-PCR

Total RNA was isolated from tissues pooled from 6 animals with the use of PureLink RNA^®^ Mini Kit (Ambion), according to the manufacturer’s instructions, and quantified spectrophotometrically by Optizen Pop Bio (Mecasys). The mRNAs obtained were reverse-transcribed into cDNAs using oligo dT and SuperScript™ II Reverse Transcriptase (Invitrogen); then, cDNA was stored at − 20 °C until use. The qPCR was performed in 10 µL with a primer concentration of 1 μM, 10 ng cDNA and 1× SYBR Green Qpcr Master Mix (EURx) and carried out in the BIOER Line-Gene K PCR. The amplification setup consisted of an initial denaturation step at 95 °C for 2 min and 40 cycles of denaturation at 95 °C for 5 s, annealing at 66 °C for 30 s and extension at 72 °C for 30 s. Samples were analysed in triplicate on separate reactions to avoid technical measurement errors. Primer pairs used for qPCR analyses were designed by using the Primer3 software (version 4.1.0 [79]) [80]. Primers sequences are reported in Table 2. The relative expression levels for each gene were calculated by the 2^−ΔΔCT^ method, and normalized using the relative expression of GAPDH.

### 4.3. Cloning and Production of Recombinant Proteins

Coding sequences for α-, β- and γ-syns were obtained by PCR on an aliquot of the same cDNAs employed for qRT-PCR. Primer sequences are reported in Table 3. *Xenopus* α-, β- and γ-syn isoforms were cloned BamHI-EcoRI in pGEX-2T to produce GST-fusion proteins. All plasmids were sequence-verified before transformation into *E. coli* BL21(DE3) cells that were grown in LB medium supplemented with ampicillin to OD600 0.5–0.6 when GST-syn expression was induced with 0.1 mM IPTG at 37 °C for 2–3 h. Cells were harvested and stored frozen at −80 °C until use. Cells were resuspended in lysis buffer (25 mM Mops pH 7, containing 150 mM NaCl, 1 mM PMSF, 1 mg/mL lysozyme) and sonicated to obtain a lysate that was clarified by centrifugation at 20,000× *g* for 20 min. All GST-syn isoforms were purified on GSH-Sepharose Fast Flow (GE Healthcare, Chicago, IL, USA) according to the manufacturer’s instructions. To remove the GST tag, the purified fusion protein was treated with thrombin (GE Healthcare, Chicago, IL, USA) for 2 h and repurified on GSH-Sepharose. Syn was recovered in the unbound and wash fractions. The purified protein was concentrated by ultrafiltration with Vivaspin10K filters (Sartorius). Protein content was measured with the microBCA assay and spectrophotometrically. CD spectra were recorded on a Jasco J-810 spectropolarimeter in the range 260–190 nm, with 0.1 cm cuvettes. All spectra are the average of at least 4 scans with buffer subtracted. For CD analyses, the proteins were exchanged in 10 mM potassium phosphate buffer pH 7, containing 50 mM Na_2_SO_4_ to remove chloride. Fluorescence spectra were recorded on a Fluoromax Jobin Yvon spectrofluorimeter at 20 °C with a 0.4 × 1 cm cuvette (excitation along the 0.4 cm path-length). Excitation was at 270 nm, emission spectra were collected between 280 and 500 nm, and the excitation and emission slit width was 5 nm.

### 4.4. Western Blot Experiments

Samples previously collected and constituted of tissues pooled from 6 animals were homogenized in a denaturing lysis buffer containing 30 mM Tris/HCl (pH 7.4), 1.5% sodium dodecyl sulphate (SDS, *w*/*v*), 8 mM EDTA (*v*/*v*) and 50 mM dithiothreitol (DTT, *v*/*v*) [41] and protease inhibitors (Roche, Indianapolis, IN, USA); then, the particulate matter was removed by centrifugation at 14,000× *g* for 20 min. The protein concentration was determined by the Bradford assay. For SDS-PAGE analysis, proteins were denatured by boiling in Laemmli Sample Buffer for 5 min. Then, 50 μg of protein was loaded in each lane and separated in 15% SDS-polyacrylamide gels according to Laemmli [81]. After electrophoresis, gels were transferred to nitrocellulose paper (Hybond C+ Extra, GE Healthcare, Chicago, IL, USA) and membranes were stained with Ponceau S to confirm the transfer of proteins. The saturation step was performed by incubating membranes in 5% bovine serum albumin (BSA) in TBS-Tween for 2 h at room temperature [35]. Then membranes were incubated overnight in the anti-α syn primary antibody ab27766 (abcam, Cambridge, UK) diluted at 1:1000, and for 1 h at room temperature with the HRP-conjugated anti-mouse secondary antibody (Sigma-Aldrich Cat# A9044). Detection was done using the Westar μC Ultra enhanced chemiluminescent HRP substrate (Cyanagen, Bologna, Italy) and Kodak X Omat LS films (Sigma-Aldrich, St. Louis, MO, USA). 

### 4.5. Immunohistochemical Analysis

Samples were fixed by immersion in PFA fixative (4% paraformaldehyde in 0.1 M phosphate buffer), pH 7 at 4 °C for 24 h, then stored at 4 °C in 0.01 M phosphate buffer (PB) containing 15% of sucrose, embedded in PB containing 10% gelatin and frozen. Samples were frozen and cut on a cryostat (HM 505 E, Microm, Walldorf, Germany) into 30-µm-thick coronal serial sections that were stored until use in 24-well plates containing cold 15% sucrose PB. Sections were enumerated to avoid misplacement, maintaining the seriality. Before immunohistochemical staining, the free-floating sections were treated with 0.01 M phosphate-buffered saline (PBS) containing 0.3% Triton X-100 (PBST) at 4 °C for 2 or 3 days to improve tissue permeability. Sections were pre-treated for 1 h at room temperature with PBST containing 0.1% sodium azide and 0.5% H_2_O_2_. To avoid the non-specific antibody binding, sections were pre-incubated with normal horse serum (Vector Laboratories, Newark, NJ, USA) diluted 1:50 in PBST, containing 1% bovine serum albumin (BSA, Sigma-Aldrich, St. Louis, MO, USA). Free-floating sections were then incubated for 5 days at 4 °C with ab27766 antibody (abcam, Cambridge, UK) dilution 1:10,000. Sections were then incubated for 1 h at room temperature with biotinylated horse anti-mouse immunoglobulin (Vector Laboratories) and then incubated for 45 minutes at room temperature with avidin-biotin-peroxidase complex (ABC, Elite Kit; Vector Laboratories, Newark, NJ, USA) diluted 1:2000 with PBST. The peroxidase activity was evidenced by a reaction with a solution containing 0.04% of 3.3-diaminobenzidine-tetrahydrochloride (DAB, Fluka, Buchs, Switzerland), 0.4% of nickel ammonium sulphate, and 0.003% of H_2_O_2_ in 0.5 M Tris-HCl buffer, pH 7.6 for 3 minutes at room temperature. Some sections were counterstained with the Nuclear Fast Red (Kernechtrot) solution (Sigma-Aldrich, St. Louis, MO, USA) after the immunohistochemistry procedure. For control experiments, the primary antiserum was substituted with buffer or normal rabbit serum. None of the control sections showed positive immuno-staining. The stained sections were mounted on glass slides maintaining the seriality, dehydrated, cleared, and cover-slipped with Permount (Fisher Scientific, Pittsburgh, PA, USA).

### 4.6. Preparation of Figures

Microsoft Power Point and Corel Draw software and BioRender [82] were used for the preparation of the figures.

## 5. Conclusions

**Figure 9:**
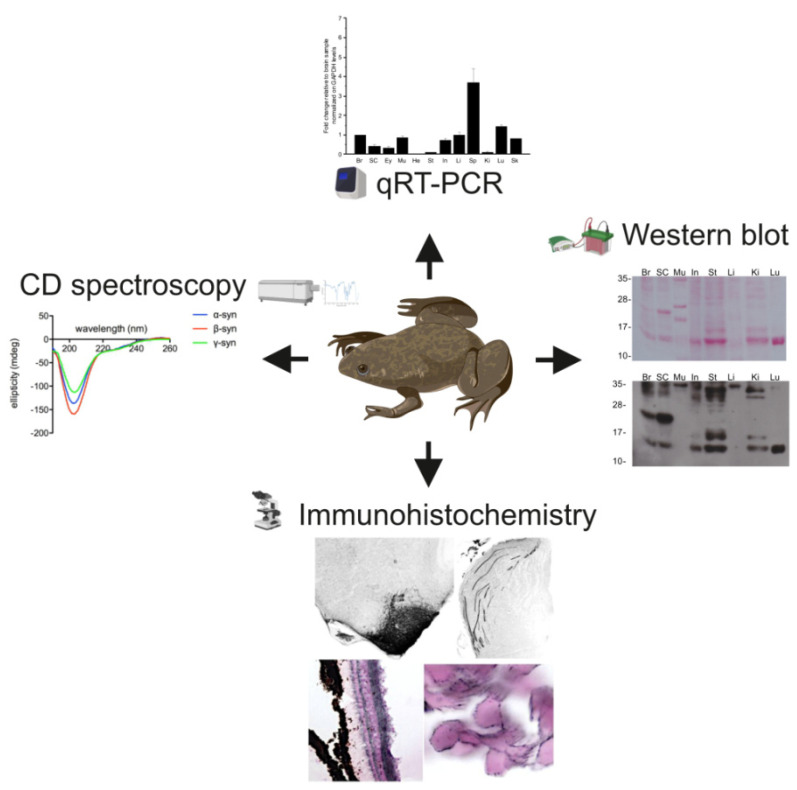
*Xenopus laevis*: a potential model for the study of synucleins.

The analysis of the sequence, secondary structure, expression and distribution of α-syn in *Xenopus laevis* (Figure 9) suggests that this amphibian, historically widely used in scientific research, may constitute a good model for the study of synucleinopathies.

## Figures and Tables

**Table 1:** mRNA coding sequences and amino acid sequence comparison between L and S syns. * indicates partial sequence.

	mRNA Coding Sequence	Amino Acid Sequence
L α-syn vs S α-syn *	95.23%	94.66%
L β-syn vs S β-syn	93.53%	92.48%
L γ-syn vs S γ-syn	99.49%	99.22%

**Table 2:** Oligonucleotide sequences used as qPCR primers for the analysis of syn transcripts.

Primer Name	Sequence (5′–3′)
*Snca*_Xn_F	CGGCCCAGAAGGGACAATGG
*Snca*_Xn_R	TCCTCCTCAGGCGGCATTTC
*Sncb*_Xn_F	GTTCCCTACAGACATCAAGCCTG
*Sncb*_Xn_R	GGGGCCTCTTCATAGTTCTCCC
*Sncg*_Xn_F	GATCTACATCCAGATCAGCCAG
*Sncg*_Xn_R	CCGACCTGCTCAATGCTTTCTGT
*Gapdh*_Xn_F	GCTGGTGCCGTGTATGTGGTG
*Gapdh*_Xn_R	CACCTCCCTTCAAGTGCAGAGA

**Table 3:** Oligonucleotide sequences used for cloning of syn coding sequences. Restriction sites used for cloning in pGEX-2T are underlined.

Primer Name	Sequence (5′–3′)
*Snca*_Xen_F	CTGGATCCATGGATGTATTCATGAAAGG
*Snca*_Xen_R	CTGAATTCTCATGCTTCAGGATCATAATCTTG
*Sncb*_Xen_F	CAGGGATCCATGGATGTGCTTATGAAAGG
*Sncb*_Xen_R	CTGAATTCTTATGCTTCAGGTTCATATTCC
*Sncg*_Xen_F	CTGGATCCATGGATGTGTTTAAGAAAGGTT
*Sncg*_Xen_R	CTGAATTCTTAATTCTCTCCATCACCGACC

## Supplementary Materials

The following supporting information can be downloaded at: www.mdpi.com/article/10.3390/ijms23116058/s1.
