# Supplementary material for: Synuclein Analysis in Adult Xenopus laevis
Source: Int J Mol Sci. 2022 May 27;23(11):6058. doi: 10.3390/ijms23116058 (PMC9181771; doi:10.3390/ijms23116058)
Supplement: Supplementary file 1 [file ijms-23-06058-s001.zip › ijms-1697179-supplementary.pdf]

a

```
αL  GATAGGTGTGGTTTCTCTGGTACCTGCTTTAAATGCTTTGATTCCATCAGGTTGTTCCATATGCACATACCTCTCAGGGGATTTATTTGGCAGTTATGTACTGAAGAGGAGACTTGATTC      120
αS  ------------------------------------------------------------------------------------------------------------------------      0

αL  CTTGTTTATTGTTTATACAAGCACAGATATCATTCCTCTTGGACTAAATTTGGGGACCAAAGGTTAATAACCGCTAGAATAAATTTATTAGCTATGGATGTATTCATGAAAGGGCTTTCC      240
αS  ------------------------------------------------------------------------------------------------------------------------      0

αL  AAAGCTAAAGAGGGTGTTGTGGCAGCTGCAGAAAAAACCAAACAGGGAGTGGCAGAAGCAGCAGGAAAGACAAAAGAGGGTGTTCTCTATGTGGGTTCAAAAACAAAGGAAGGTGTTGTA      360
αS  -AAGCAAAAGAGGGTGTTGTGGCAGCTGCAGAAAAAACCAAACAGGGAGTGGCAGAAGCAGCAGGAAAAACAAAAGAGGGTGTTCTCTATATGGGCTCAAAAACAAAGGAAGGCGTTGTA      119
     **** ************************************************************ ******************** **** **************** ******

αL  CATGGAGTAACAACAGTTGCTGAGAAGACCAAAGAGCAGGTGTCTAATGTTGGTGGAGCAGTAGTTACAGGGGTAACTGCAGTTGCACACAAGACAGTAGAAGGAGCCGGTAATTTTGCT      480
αS  CATGGAGTAACAACAGTTGCGGAGAAGACCAAAGAACAGGTGTCTAATGTTGGTGAAGCAGTAGTTACAGGAATGACTGCAGTTGCACAGAAGACAGTAGAAGGAGCTGGAAATATTGCT      239
    ******************** ************** ******************* ***************  * ************** ***************** ** *** *****

αL  GCAGCAACAGGCCTGGTAAAGAAGGATCAAAAGAATGAAAGCGGCTTCGGCCCAGAAGGGACAATGGAAAATTCAGAAAATATGCCGGTAAATCCAAATAATGAGACATATGAAATGCCG      600
αS  GCAGCAACAGGCCTGGTAAAGAAGGATCAAAAGAATGAAAGCGGCTTCGGCCCAGAAGGGACAGTGGAGAATTCAGAAAATATGCCGGTAAATCCAGATAATGAGACATATGAAATGCCG      359
    *************************************************************** **** **************************** **********************

αL  CCTGAGGAGGAATATCAAGATTATGATCCTGAAGCATGAAAAATCAGCGCAAATTACATCCTTTAGAAGATTCTATTTCATATGAAAATGTGTTCTAATGTAGCCATTTGTAATTTTTTT      720
αS  CCTGAGGAGGAGTATCAAGATTATGATCCTGAAGCATGAATAATCAGCGCAAATTACATCCTGTAGGAGATTCTATTTCATATGAAAATGTGTTCCAATGTAGCCATTTGTCATTTT---      476
    *********** **************************** ********************* *** **************************** *************** *****

αL  TTTTGGATATTTTCTCAGTTGTTTATTAAGTCTTCCATCCACAGTGATAAAGGCATCACAAAATAGGGTTAATTATCAGAA-------------------CTAGTGACCCAATGACTCCA      821
αS  -TTTGTATATTTCTGCAGTTGTTTATGAAGTCTTACATCCACAGTGATAAAGACATCACAAAATAGGGTTAATTATCAGAAACCTATTATCATCAGATAATCAGAGACCCATTAACTCCA      595
     **** ******   *********** ******* **************** ****************************                      ** ****** * ******

αL  ACCTACTAAAATGGGAAGAATTGGTAGGGGATTTTTTGTGGATGTTGTGACTTCAATTTTCAAACAAAATTGATTAAAAACA-----G-AAAAAAGAAAGAAAAACCACCTAAGTGTCTA      935
αS  CCCTACTAAAGCGGGCAGAATTTAA-----------------------------ATTTTAAAAACAATATTGATTTAAACAAAAAAAGAAAGAAAGAAAAAAAAAACACCTAAGTGTCTA      686
    *********  *** ******                               * ***  ****** ******* ***  *     * ** ******* ***** *************

αL  CTATTGTCTTACTTTCTATTTGTATGAT----TGTTTTCAGTGGT-TTGGTATTGTTTATAAAAATTTTGTATG-TTTCCTATGTCCACTACTGTTACATGTAACATATTGTGATACCAG      1049
αS  CTAATGTCTTACTTTCTATTTGTATGATTGACTGTTTTCAGTGGTTTTGGTATTGTTTATAATAATTTTGTATGGTTTCCTACGTCCTCTACTGTTACATGTAACATATTGTGGTACCAG      806
    *** ***********************    ************* *************** *********** ******* **** ************************* ******

αL  TTTCTAGCTATACAGTATTTCTG-------GCATGATCTATGCACAAAGCAGTTTTACATTTTTATCATGCACCCT-ATTGATTTTGTGTTTATAATGAAATGTGTTGCTATTTAAAGAA      1161
αS  TTTCTAGCTATACGGTATTTCTGACAGTAAGCATGATCTATGCACCAAGCAATTTTACATTTCTATCATGCACCCTGATTGATTTTGTGTTTATAATGAAATGTATTGCTATTTAAAGC-      925
    ************* *********       *************** ***** ********** ************* ************************** ************

αL  ATAAAAATAGAGTCAATAAAGATTAACATTTATGTTAAATGCAAAAAAAAAAAAAAAAAAAAAAAAAAAAAA 1233
αS  ------ATAGAGTCAATAAAGATTAATATTTATGTTAAATGCAAAAAAAAAAAAAAAAAAAAAAAAAA----- 986
          ******************** ***************************************
```

b

```
αL  MDVFMKGLSKAKEGVVAAAEKTKQGVAEAAGKTKEGVLYVGSKTKEGVVHGVTTVAEKTKEQVSNVGGAVVTGVTAVAHKTVEGAGNFAAATGLVKKDQKNESGFGPEGTMENSENMPVN      120
αS  ----------AKEGVVAAAEKTKQGVAEAAGKTKEGVLYMGSKTKEGVVHGVTTVAEKTKEQVSNVGEAVVTGMTAVAQKTVEGAGNIAAATGLVKKDQKNESGFGPEGTVENSENMPVN      110
              ****************************:************************** *****:****:********:*********************:*********

αL  PNNETYEMPPEEEYQDYDPEA   141                                                                α-syn L MW: 14736.39; pI: 4.91
αS  PDNETYEMPPEEEYQDYDPEA   131
    *:*******************
```

Figure S1. mRNA and amino acid sequence of *Xenopus* synucleins available at the NCBI database. The nt sequences of L and S α-syn mRNA are reported in A. The coding regions are represented in bold. The regions against which the forward and reverse RT-PCR primers were designed are highlighted in grey. The deduced aa sequences of L and S α-syn are reported in B. The predicted MW and pI are indicated. The currently available S α-syn mRNA sequence is partial. The sequences were aligned with Clustal Omega at https://www.ebi.ac.uk/Tools/msa/clustalo/. Asterisks indicate identity of amino acids; double dots indicate amino acids with the same polarity or size; dots indicate semiconserved substitutions.

a

```
βL  ------------------------------------------------CTGTGAGCTCTGCATCTGAGCCCTCCTCTGCCTCATCTCCACACTGAGGAAGAAGAGAGGCAGAATCTCCT     71
βS  GAAGCATCCCATGGCTCTGTAGCACTGACACTGACTGCCGAGGGACCCCCTGTGAGCTCTGTATCTGAGCCCTCCTCTGCCTCATCTCCACACTGAGGAAGAAAAGAGGCAGAATCTCGT     120
                                                    ************ ***************************************** ************* *

βL  CTGAAGCCCAGGCAGCTTATTT-TGCACAGGACACTGTTGCTGCACAAAGAGAGGTGTTGGAAGACTGCAACAGATCAGAATGGATGTGCTTATGAAAGGCTTTTCCAAAGCAAAGGAAG     190
βS  CTGAAGCCCAGGCAGATTGTTTTAGCACAGGACGCTGTTACTGCAAATAGAGAGGTGTTGAAAGACTGCAACAGATCAGAATGGATGTGTTTATGAAAGGCTTTTCCAAAGCAAAGGAAG     240
    *************** ** ***  ********* ***** ***** * ************ **************************** ******************************

βL  GTGTGGTGGCTGCAGCCGAAAAGACTAAGCAAGGTGTAGCAGAGGCTGCAGAGAAAACCAAGGAAGGTGTCCTATACATGGGAAGCAAAACCCGAGATGGAGTGGTGCAAGGAGTGACTT     310
βS  GTGTGGTGGCTGCAGCCGAAAAGACTAAGCAAGGGGTAGCAGAGGCTGCAGAGAAAACCAAGGAAGGTGTCCTTTATGTGGGGAACAAAACCCGAGATGGTGTGGTGCAAGGAGTGACTT     360
    ********************************** ************************************** **   **** * ************** ******************

βL  CAGTGGCTGAAATGACCAAAGAGCAGGCATCCCAGCTTGGAGGAGCTGTCATGTCTGGAGCTGGGAACATTGCTGCAGCTACTGGACTGGTGAAGAAGGATGAGTTCCCTACAGACATCA     430
βS  CAGTTGCTGAAAAGACCAAAGAGCAGGCATCCCACCTTGGGGGAGCCGTCATGTCTGGGGCTGGGAACATCGCTGCAGCTACTGGACTGGTGAAAAAGGATGAGTTCCCTACAGACCTCA     480
    **** ******* ******************** ***** ***** *********** *********** ********************** ******************** ***

βL  AGCCTGAAGAAGAGGGCCAGGAAGCTTTGGAAGAACCTGCAGCTGAGCCTCTCCTGGAACTAGAAGGGGAGAACTATGAAGAGGCCCCACAGGATGATTACCAGGAATATGAGCCTGAAG     550
βS  AGCCTGAAGAAGAAGCCCAAGAAGCTTTGGAAGAACCTGCAGCTGAGCCACTCTTGGAACCTGAAGGGGAGAGCTATGAAGA---CCCACAGGATGATTACCAGGAATATGAACCTGAAG     597
    ************ * *** **************************** *** ******  ********** *********   **************************** *******

βL  CATAACAACACAATATTCTGGGCCCCTCCACCAGCAACATCAGCACAAAGATAGCTATAATGAATAAAAAAAAAATACAAATAAAGTTCCTTTTCCTAAGCCCCCCCCATTTCTTTTAAAA     670
βS  CATAACAACCCCATATTTTGGGTCCCTCCACCAGCAACATCAGCACAACGATCGCTATAATGAAAAA--TAAAATAAAAACAAAGTTCCTTTTTCCTACGCCCTCCTACTTCCTTTCAAA     715
    ********* * ***** **** ************************ *** *********** **    ****** *** ************ *  *  *** ** * *** *** ***

βL  AGATGGATGAAGCCCACCTCCCTCTTCTCCTAAGAAGTGACTTCTCCCTTCAATTCACCTGCAGAGAACCTCATGTGAAGTGATTTTATGTATGGTAACCCTTAGCGTTGAGTCATAGTC     790
βS  A-------GAAGCCCATCTCCCTCTTCTCCTAAGAAGTGACTTCTTCCTTCA-GTCACCTGCAGAGAACCCCGTGTGAAGTGATTTTATGTATGGTAACCCTTAGCATT--GTCATAGTC     825
    *       ******** ************************** ******  *************** * ********************************* **  *********

βL  CAAGCAAAAATGGATGTTTAAAACAAAGAAAA-AAAAGAATCCCTTGATAATGTACGGAAACCATGAATGCATTGTTACTGTTTTTTGCAGTCCTTTTTGCCCCCGAGGAATTATTTCTG     909
βS  CAATCAAAAATGGTTGTTTAAAACAAAGAAAAAAAAAGAATCCCTTGATAATGTATGAAAATCATGAATGCACTGTTACTGTCTTTTGAAGTCCTT-TTGCCCCCGAGGAATTATTTCTG     944
    *** ********* ****************** ********************** * *** ********** ********* ***** ******* **********************

βL  TTTCCCTCCCAGAGAATAATTGTCGGGGGTTCAGTGAATTTTGTGTCCTATATTTATTTTGTAACTTGTTTATTGTTTTTTTCTCTTTTGCTCTGACGTAATAGCTGATTCTGTGCCAGG     1029
βS  CTTCCCTCCCAGAGAATAATTGTCGGGGGTTCAGTGAATTTTGTGTCTCAAATTTATTTTGTAAGTTGTTT------TTTTTCTCGTTTGTTCTGACGTAATAGCTGATTCTGTGCCAGG     1058
     ********************************************  * ************* ******      ******** **** ******************************

βL  GGGGCCTGCAACTCCTTGCAACCGACCCAGCTAATGCTACAGGGTGTCTACCTTCATAAAATAAGCATGTTTTATCTTTTTATATACATATGGTGCACAAACACCACCAAAAAAAACCTT     1149
βS  GGGGCCTGCAACTCCCTGCACCTGGCCCAGCTAATGCTACAGCCTGTCTACCTTCATAAAATAAGCATGTTTTATCTTTTTATATACATATGGTGCACCAACACCACCAAAAACT-ACTT     1177
    ************** **** * * *****************  ******************************************************* *************    ***

βL  TTCTCTTTAACTACTCATTTGCCAAGAATTTGCACGAGGCGGCATCTTTTGGGCATTTGTGGAGTTAAA-----ACCAAAAAAAAATGTAGCTGTGGATGACTGACCTTTTGACATAGTTA     1264
βS  TTCTGTTTAACTTCTCATTTGCCAAGAATTTGCACAAGGC-GCATCTTTTGGGCATTTGTGGACTTAGACTGGAAACCAAAAAAAATTTAGTTGTGGATGACTGACCT-TTGACATAGTTA     1295
    **** ******* ******************** **** ********************** *** *     * * ******** *** **************** ***********

βL  AATCAGTGTTATAATGTGACAGTTCTCTCTCTTTTTTTGCTGTTGTTTTTT------TATACATATCTACAAATATATATCTGGTTTAACTTATTTGTGATTGTTCTGTAAAGCAGAGAG     1378
βS  AATCAGTGTTATAATGTGACAGTTCTCTCTCTTTTTTTGCTGTTGTTTTTATACATATACATATCTACAAATATATATCTGGTTTAACTTATTTGTGACTGTTCTGTAAAGCAGAGAG     1415
    ***************************************************       ***************************************** ******************

βL  AAATATGTGTGAATCCATACAGAATCTTTTGAATGTTCTTGTGTTCCAAATAATTTTGCAAAATGAATAAAAAA---AATAGGATTAACCAAAAAAAAAAAAAAAAAAAAAAAAAAAAAA     1495
βS  AAATCTGTGAGAATCCATACAGAATCTTTTGAATGTTCTTGTGTTCCAAATAATTTTGCAAAACGAATTAAAAAAGAAAAAGGATAAACCAAAAAAAAAAAAAAAAAAAAAAAAAAAAAA     1535
    **** **** ***************************************************** **** *****   ** ***** *******************************

βL  AAAAAAAAAAAAAAAAAAAAAAAAAAAAAAAAAAAAAAAAAA1537
βS  A-----------------------------------------1536
    *
```

b

```
βL  MDVLMKGFSKAKEGVVAAAEKTKQGVAEAAEKTKEGVLYMGSKTRDGVVQGVTSVAEMTKEQASQLGGAVMSGAGNIAAATGLVKKDEFPTDIKPEEEGQEALEEPAAEPLLELEGENYE     120
βS  MDVFMKGFSKAKEGVVAAAEKTKQGVAEAAEKTKEGVLYVGNKTRDGVVQGVTSVAEKTKEQASHLGGAVMSGAGNIAAATGLVKKDEFPTDLKPEEEAQEALEEPAAEPLLEPEGESYE     120
    ***:*********************************:*.************** ******:**************************:*****.************* ***.**

βL  EAPQDDYQEYEPEA 134                                                                 X b-syn L MW: 14197.73; pI: 4.29
βS  -DPQDDYQEYEPEA 133                                                                 X b-syn S MW: 14118.55; pI: 4.39
      ************
```

Figure S2. mRNA and amino acid sequence of *Xenopus* synucleins available at the NCBI database. The nt sequences of L and S β-syns mRNA are reported in A. The coding regions are represented in bold. The regions against which the forward and reverse RT-PCR primers were designed are highlighted in grey. The deduced aa sequences of L and S β-syn are reported in B. The predicted MW and pI are indicated. The sequences were aligned with Clustal Omega at https://www.ebi.ac.uk/Tools/msa/clustalo/. Asterisks indicate identity of amino acids; double dots indicate amino acids with the same polarity or size; dots indicate semiconserved substitutions.

a

```
γL  -GACCACACTGCTACTACCCTTCAGGACCGGTGCATCTTCTCAAGAGTGACACACACAAAGTTGAGGACCCCCAAGTCTTGCAGAAAGCAAACAACCATGGATGTGTTTAAGAAAGGTTT     119
γS  TGAGTGG-ATCCAAATATTCGGTCGAGCCGGTGC-TCTTCTCAAGAGTGACACACACAAAGTTGAGGACCCCCAAGTCTTGCAGAAAGCAAACAACCATGGATGTGTTTAAGAAAGGTTT     118
     **      * * * **  *    *  ******* *************************************************************************************

γL  TTCTATGGCTAAAGAAGGCGTGGTTGCTGCAGCAGAGAAAACCAAGCAGGGTGTGACAGAAGCTGCAGAAAAAACCAAGGAGGGGGTCATGTATGTAGGAGCGAAAACTAAAGAGGGTGT     239
γS  TTCTATGGCTAAAGAAGGCGTGGTTGCTGCAGCAGAGAAAACCAAGCAGGGTGTGACAGAAGCTGCAGAAAAAACCAAGGAGGGGGTCATGTATGTAGGAGCGAAAACTAAAGAGGGTGT     238
    ************************************************************************************************************************

γL  TGTACACAGTGTGAATACAGTTGCAGAGAAAACCAAAGAACAGGCCAATGTGGTTGGTGGAGCCGTGGTTTCTGGAGTAAATCAAGTATCTTCAAAGACTGTAGAAGGCACAGAGAATGT     359
γS  TGTATACAGTGTGAATACAGTTGCGGAGAAAACCAAAGAACAGGCCAATGTGGTTGGTGGAGCCGTGGTTTCTGGAGTAAATCAAGTATCTTCAAAGACTGTAGAAGGCACAGAGAATGT     358
    **** ****************** ************************************************************************************************

γL  TGTAAGCTCTACTGGTTTAGTAAAAAAGGAAGATCTACATCCAGATCAGCCAGAAGAACCTGCTGCAGAAGAACCCGCAGTGGAGGCCACAGAAAGCATTGAGCAGGTCGGTGATGGAGA     479
γS  TGTAAGCTCTACTGGTTTAGTAAAAAAGGAAGATCTACATCCAGATCAGCCAGAAGAACCTGCTGCAGAAGAACCCGCAGTGGAGGCCACAGAAAGCATTGAGCAGGTCGGTGATGGAGA     478
    ************************************************************************************************************************

γL  GAATTAATATTTCACCCTGTGTGCTGCTGGGAATCTTTGAAAAACTAGCTAAGGACATAGAAAAGAAGTAATTCATACCACAAGAATACCACAGGCTGAAAGCCAAACAACGCGTGAAAT     599
γS  GAATTAATATTTCACCCTGTGTGCTGCTGGGAATCTTTGAAAAACTAGCTAAGGACATAGAAAAGAAGTAATTCATACCACAAGAATACCACAGGCTGAAAGCCAATCAACGCGTGAAAT     598
    ********************************************************************************************************** *************

γL  TTTCCTCTTGAATTATCAAGTCCGTCAGTGTATGCATTCATGTTATAATTGTGAATGTACAGCTTTGTAACCCAACCGATATACCACCAGTCACACATCCTTCACATCTTCATGTGATTT     719
γS  TTTCCTCTTGAATTATCAAGTCCGTCAGTGTATGCATTCATGTTATAATTGTGAATGTACAGCTTTGTAACCCAACCGATATACCACCAGTCACACATCCTTCACATCTTCATGTGATTT     718
    ************************************************************************************************************************

γL  ATTATACACAACTTTGTTTGACCCTGAGCTCTGGTTTTACTAACAGTCTATTTTTACCCCTATCAATCTTCATTTGTATGTGTTTTTATTTGACGCACCTACTACTGATCCAGTGCTCTG     839
γS  ATTATACACAACTTTGTTTGACCCTGAGCTCTGGTTTTACTAACAGTCTATTTTTACCCCTATCAATCTTCATTTGTATGTGTTTTTATTTGACGCACCTACTAATGATCCAGTGCTCTG     838
    ******************************************************************************************************** ***************

γL  CATTACTCCATCTATGAATAGCTCTCCTACTCAAGTACCCTATTGTATCAACTCCTTTTTTTCTTGTTAGTTTTTGCTTGGGTCACCATTCAGCATCATCTTTATATTCACATTGGCTAT     959
γS  CATTACTCCATCTATGAATAGCTCTCCTACTCAAGTACCCTATTGTATCAACTCCTTTTTTTCTTGTTAGTTTTTGCTTGGGTCACCATTCAGCATCATCTTTATATTCACATTGGCTAT     958
    ************************************************************************************************************************

γL  ATTGTCCCTTCTTAAAGCTATTTTTTTTTTTCAGCCATACAAATAAAACTTTATTTGACAAAAAAAAAAAAAAAAAAAAAAAAAAAAA  1046
γS  ATTGTCCCTTCTTAAAGCTATTTTT-TTTTCAGCCATACAAATAAAACTTTATTTGAGTAAAAAAAAAAAAAAAAAAAAAAAAA----  1040
    ************************* *******************************   ***********************
```

b

```
γL  MDVFKKGFSMAKEGVVAAAEKTKQGVTEAAEKTKEGVMYVGAKTKEGVVHSVNTVAEKTKEQANVVGGAVVSGVNQVSSKTVEGTENVVSSTGLVKKEDLHPDQPEEPAAEEPAVEATES     120
γS  MDVFKKGFSMAKEGVVAAAEKTKQGVTEAAEKTKEGVMYVGAKTKEGVVYSVNTVAEKTKEQANVVGGAVVSGVNQVSSKTVEGTENVVSSTGLVKKEDLHPDQPEEPAAEEPAVEATES     120
    *************************************************:**********************************************************************

γL  IEQVGDGEN  129                                                                    X g-syn L MW: 13415.88; pI: 4.75
γS  IEQVGDGEN  129                                                                    X g-syn S MW: 13441.91; pI: 4.66
    *********
```

Figure S3. mRNA and amino acid sequence of *Xenopus* synucleins available at the NCBI database. The nt sequences of L and S γ-syns mRNA are reported in A. The coding regions are represented in bold. The regions against which the forward and reverse RT-PCR primers were designed are highlighted in grey. The deduced aa sequences of L and S β-syn are reported in B. The predicted MW and pI are indicated. The sequences were aligned with Clustal Omega at https://www.ebi.ac.uk/Tools/msa/clustalo/. Asterisks indicate identity of amino acids; double dots indicate amino acids with the same polarity or size; dots indicate semiconserved substitutions.
